# Mechanisms of Metal-Induced Mitochondrial Dysfunction in Neurological Disorders

**DOI:** 10.3390/toxics9060142

**Published:** 2021-06-17

**Authors:** Hong Cheng, Bobo Yang, Tao Ke, Shaojun Li, Xiaobo Yang, Michael Aschner, Pan Chen

**Affiliations:** 1 Department of Occupational Health and Environmental Health, School of Public Health, Guangxi Medical University, Nanning 530021, China; chenghong@stu.gxmu.edu.cn (H.C.); yangx@gxmu.edu.cn (X.Y.); 2 Department of Molecular Pharmacology, Albert Einstein College of Medicine, Bronx, NY 10461, USA; bobo.yang@einsteinmed.org (B.Y.); tao.ke@einsteinmed.org (T.K.); 3 Department of Toxicology, School of Public Health, Guangxi Medical University, Nanning 530021, China; lishaojun0613@163.com; 4 Department of Public Health, School of Medicine, Guangxi University of Science and Technology, Liuzhou 545006, China

**Keywords:** mitochondrial dysfunction, neurological disorders, metals, neurotoxicity

## Abstract

Metals are actively involved in multiple catalytic physiological activities. However, metal overload may result in neurotoxicity as it increases formation of reactive oxygen species (ROS) and elevates oxidative stress in the nervous system. Mitochondria are a key target of metal-induced toxicity, given their role in energy production. As the brain consumes a large amount of energy, mitochondrial dysfunction and the subsequent decrease in levels of ATP may significantly disrupt brain function, resulting in neuronal cell death and ensuing neurological disorders. Here, we address contemporary studies on metal-induced mitochondrial dysfunction and its impact on the nervous system.

## 1. Introduction

Mitochondria play a key role in many cellular physiological and pathological processes, including energy metabolism, calcium homeostasis, lipid biosynthesis, and apoptosis [1]. One of their main functions is to produce adenosine triphosphate (ATP) by coupling the electron transport chain (ETC) with phosphorylation. The ETC consists of four major protein–metal complexes (I–V) which primarily serve to generate a proton gradient to drive the production of ATP [2]. Superoxide anion, a byproduct of the ETC’s operation, is extremely unstable and rapidly converted into hydrogen peroxide (H_2_O_2_) and ROS in the cytoplasm [3]. However, excessive production of ROS may cause oxidative stress, ETC dysfuction, mitochondrial structural damage [4,5], and oxidative damage to proteins, DNA, and lipids [6].

Neurons are highly polarized cells, heavily dependent on the energy generated by mitochondria, and the brain consumes about 20% of the body’s resting ATP, while it accounts for only about 2% of the body’s mass [7,8]. In addition, mitochondria are necessary calcium-buffering organelles in neurons as they regulate local calcium dynamics to control neurotransmitter release [9]. Mitochondrial dysfunction has been implicated in a variety of diseases, and is a causative factor in several neurodegenerative diseases, including Alzheimer’s disease (AD), Parkinson’s disease (PD), Huntington’s disease (HD), autism, and amyotrophic lateral sclerosis (ALS) [10,11,12].

Among the chemical elements that humans are exposed to, metals play an important role in both health and disease. Metals are natural components of the Earth’s crust and enter the biosphere through a variety of human activities [13]. They are generally classified into two groups: essential and non-essential metals. The main routes of human exposure include ingestion, inhalation, and dermal contact [14]. The brain is able to regulate these metals effectively under physiological conditions. However, excessive exposure to metals, such as arsenic (As), aluminum (Al), cadmium (Cd), lead (Pb), copper (Cu), and manganese (Mn) may lead to their accumulation, and ensuing neurodegeneration [15]. Mitochondrial impairment and metal dyshomeostasis have been linked to some neurodegenerative disorders including AD, PD, HD, and ALS [12]. Metals can cause neurodegeneration by disrupting mitochondrial function, and thereby deplete ATP, induce ROS production, and ultimately lead to cell death through apoptotic and/or necrotic mechanisms [16]. There has been a growing interest in understanding the metabolism of neurotoxic metals and their role in the etiology of various neurodegenerative diseases, and a great deal of research has been done for this purpose. However, the effects of various metals on different neurodegenerative diseases are not identical, and their specific mechanisms of damage have yet to be fully clarified. Therefore, in this review, we summarize the latest reports on the mechanism of mitochondrial dysfunction in neurodegenerative diseases caused by metal exposure.

## 2. Neurological Disorders with Mitochondrial Dysfunction and Oxidative Stress

### 2.1. Alzheimer’s Disease (AD)

AD is a well-known age-related neurodegenerative disorder characterized by progressive decline in cognitive function and pathological features of increased neuronal cell death [17]. The etiological hypotheses for AD mainly include genetics [18], decreased acetylcholine synthesis [19], accumulation of neurotoxic protein plaques of amyloid-β (Aβ) peptide [20], fibrous tangles with high phosphorylation of tau protein (P-tau) [21], or irregular mitochondrial function and dynamics [2]. However, the pathogenesis of AD remains unclear. It has been demonstrated that mitochondrial dysfunction is an early event in AD pathogenesis, characterized by decreased metabolism, disruption of Ca^2+^ homeostasis, elevated ROS levels, lipid peroxidation, and apoptosis [22]. An increased association between mitochondria-associated membranes (MAM) and mitochondria has also been linked to the pathogenesis of AD [23,24]. Moreover, variations in mtDNA have also been found to be related to the pathogenesis of AD, such as mutations in the heteroplasmic somatic mtDNA control region [25] and mitochondrial point/missense mutations in genes encoding cytochrome c oxidase subunits I, II, and III [26].

Although aging is a major risk factor for AD, extensive epidemiological evidence suggests that exposure to environmental toxins, particularly pesticides, metals, and solvents, may increase the risk of developing neurodegenerative diseases [27]. Neurotoxic metals, such as Pb [28], Hg [29], Al [30], Cd [31], and As [32] have been implicated in AD due to their ability to increase Aβ peptide and P-tau phosphorylation, leading to senile/amyloid plaques and neurofibrillary tangles (NFTs). Synergistic exposure to Pb, As, and Cd has been shown to further enhance the expression of amyloid precursor protein (APP) and BACE1, which in turn maximizes the induction of Aβ production [32]. A recent in vivo study showed that chronic inorganic arsenic (iAs) exposure aggravated AD-like pathology in 3xTgAD mouse brain, including reduced ATP content and complex I levels, as well as increased ROS formation in the hippocamus. In addition, higher immunopositive responses to amyloid isoforms and phosphorylated tau were observed in the frontal cortex and hippocampus [33]. It has been proved that Al in the brain can regulate the expression, distribution, and accumulation of APP and induce the maladjustment of iron-modulated signaling pathways through its interaction with the IRE mRNA regions, thus stimulating Fe^2+^-induced membrane lipid peroxidation and causing oxidative damage [30,34,35].

A large body of evidence suggests a role for essential metal ion dysregulation in the etiology of AD, in particular the accumulation of Cu, zinc (Zn), and iron (Fe) in the amyloid plaques [36,37,38]. It is well established that an increase in loosely bound Cu and Fe in human AD brains can promote oxidative stress [39]. It is worth noting that although the total copper content in the AD brain is lower, the proportion of redox-active exchangeable Cu is higher, which is positively correlated with increased oxidative damage and AD neuropathology [40]. Similarly, tau displays redox activity when it binds to Cu, leading to further oxidative damage in the brain [41]. Mitochondrial ferritin deficiency aggravates the neurotoxicity induced by β-amyloid in mice, which may be related to the increase in intracellular iron accumulation and oxidative stress levels [42]. In vitro studies have demonstrated that binding of iron to Aβ peptide can promote Aβ aggregation and further increase the neurotoxicity of Aβ [43,44,45] by regulating the redox potential to the level at which iron’s redox cycling occurs, which not only leads to the production of oxidative species, but also consumes essential oxygen and biological reductants [46]. ROS or exogenous oxidants can promote the release of harmful zinc from metallothionin, which in turn leads to mitochondrial dysfunction and further oxidative stress [47,48,49], and affects protein aggregation [50,51,52]. Studies have shown that zinc content is particularly high in AD neurons expressing mutant APP, PSEN1, and tau [53,54]. A systematic review and meta-analysis has shown that patients with AD had lower serum Mn levels, suggesting that Mn deficiency may be a risk factor for AD [55]. However, the link between Mn and AD remains very limited. The expression level of MnSOD in the hippocampal CA1–CA4 region of AD patients was 3–11 fold higher than that of the control group, suggesting the normal compensatory mechanism of Mn-dependent antioxidant enzymes may not be sufficient to protect the hippocampus from free radical oxidative damage [56].

### 2.2. Parkinson’s Disease (PD)

PD is the second most prevalent and incidental neurodegenerative disease, affecting more than 2% of the population older than 65 years old [57]. Typical symptoms of PD include rigidity, bradykinesia, and test tremor [58]. The main pathological features of PD include selective loss of dopaminergic neurons in the substantia nigra (SN) region of the brain and more widespread aggregation of protein α-synuclein in Lewy bodies (LB) [59]. PD is accociated with mitochondrial dysfunction and calcium and dopamine (DA) dyshomeostasis, as well as abnormal autophagy and proteostasis [60].

Numerous studies have posited that mitochondrial dysfunction plays a key role in the pathogenesis of PD. The first line of evidence was documented in 1989 by Schapira and co-workers as they found a decrease in complex I of the ETC in the SN pars compacta (SNpc) of PD patients [61], which has been further confirmed [62]. The expression of mitochondrial proteins were changed, such as the molecular chaperones [63], the protease HtrA2 [64], a and b hemoglobins [65], or the outer mitochondrial membrane VDAC1 [66]. Recent studies showed that a vicious cycle between α-synuclein aggregation and mitochondrial impairment may exist in DA neurons [67]. Alterations in the PD-related genes DJ-1, PINK1, parkin, alpha-synuclein, and LRRK2 can directly or indirectly lead to mitochondrial dysfunction, resulting in increased ROS production and susceptibility to oxidative stress [68,69]. Oxidative stress plays an important role in the degeneration of dopaminergic neurons in PD [70]. Accumulating evidence has shown that oxidative stress is elevated in the brains of PD patients of both genetic and sporadic cases, and oxidative stress markers can be found in the SNpc DA neurons and their striatal axons [69,71]. DA metabolism, mitochondrial dysfunction, and neuroinflammatory processes are the main contributors to oxidative stress augmentation in PD [72].

More than 90% of PD cases are sporadic, and the etiology is associated with the complex interaction between genetic susceptibility and environmental stimuli [73]. The role of metals in the pathogenesis of PD has been the focus in medical chemistry and neurotoxicology [74,75]. Epidemiological studies have reported positive correlations between PD and long-term exposure to metals, such as Mn, Hg, Cu, Pb, Zn, Fe, and Al [76,77]. Metal exposure has been associated with key factors in the pathogenesis of PD, such as mitochondrial dysfunction, alterations in metal homeostasis, and aggregation of a-synuclein [30,78,79,80,81]. Epidemiological studies have reported a significant dose–response relationship between PD patients and blood Hg levels [82]. Hg has been found to cause neuron loss and cognitive and motor impairments in animal models, and further in vitro studies have shown that mercury exposure can cause apoptosis and oxidative stress [83,84,85]. SN neurons contain neuromelanin that can bind Fe and generate free radicals, causing cell death and lipid peroxidation [86]. Fe can also induce dopamine oxidation in SN neurons, which leads to the release of additional free radicals [87]. The binding of Cu to α-synuclein can induce oxidative damage of the protein and the oxidation of some C-terminal residues can promote protein aggregation [88,89,90,91]. High concentrations of free Zn in the PD anterior olfactory nucleus was detected and the colocalization of free zinc and alpha-synuclein suggested the role of zinc in the pathogenesis of PD [92]. In a mouse model of PD, Mn exposure can enhance mitochondrial dysfunction to aggravate neurodegeneration and progressive motor deficits [93].

### 2.3. Huntington’s Disease (HD)

HD is an inherited autosomal dominant neurodegenerative disorder caused by a CAG amplification of the huntingtin (Htt) gene. The typical clinical manifestations are chorea-like involuntary movements, dementia, and psychiatric symptoms [94], and the pathological features are selective loss of striatal neurons and aggregation of the mutant Htt protein [95,96]. Disruptions of mitochondrial energy metabolism were found in the brains of patients with advanced HD, including reduced activities of mitochondrial respiratory complexes II–IV and aconitase [97,98]. In addition, in vitro studies with samples from presymptomatic and pathological Grade 1 HD patients showed no changes in striatal or cortical complexes I–IV activity, suggesting that mitochondrial energy impairment is a late event in the progression of the disease, rather than a cause [99,100]. HD patients also exhibit weight loss, which may be due to the mitochondrial ATP synthesis disorder [101]. The pathogenesis of HD is related to mitochondrial dysfunction, which is manifested by reduced ATP/ADP ratio, decreased O_2_ consumption, increased mitochondrial ROS and fragmentation, abnormal lactate/pyruvate levels, and decreased mitochondrial membrane potential [102]. These apparent mitochondrial dysfunctions may be related to interaction with mutant Htt [103]. Mutant Htt can interfere with mitochondrial function by binding to Drp1 to disrupt the balance of mitochondrial fission–fusion dynamics, reducing anterograde and retrograde axonal mitochondrial transport, and binding to peroxisome proliferator-activated receptor coactivator-1α (PGC-1α) protein which is involved in mitochondrial biogenesis and antioxidant defenses [104,105]. The ultimate result of these mitochondrial injury is a reduction in ATP production, with ensuing neuronal dysfunction followed by death [106].

Although evidence of metal involvement in HD pathogenesis is limited, histological and MRI studies demonstrate elevated basal ganglia iron levels in HD patients [107]. Agrawal and Fox found that mitochondrial iron accumulated in a mouse model HD brain, and neonatal iron supplementation could increase the accumulation of mitochondrial iron in the brains and enhanced markers of mitochondrial dysfunction [108]. Cu has been shown to promote aggregation of huntingtin protein [109]; however, it is not clear whether the abnormal distribution of Cu interacts with mitochondria [59]. The HD-associated mitochondrial inhibitor 3-nitropropionic acid (3-NPA) causes Zn accumulation in vitro or in vivo [110]. Studies have found that Mn deficiency is related to HD [111,112,113,114,115,116], and exogenous Mn supplementation can promote the clearance of mutant HTT protein aggregates in striatum cells [117,118]. A recent study reported that Mn-induced mitochondrial dysfunction in HD cells could only be detected at an exposure dose above the acute toxicity threshold [119]. One study found that Cd exposure increased oxidative stress, caused apoptosis, and altered metal transport in heterozygous HTT striatum cells [120].

### 2.4. Autism

Autism spectrum disorder (ASD) is a complex neurodevelopmental disorder characterized by impairments in reciprocal social interaction and communication, as well as restricted and stereotyped patterns of interests and behaviors [121]. The pathogenesis of ASD is unclear, and its clinical manifestations are varied. The etiology of ASD may involve a variety of genetic and environmental factors [122]. Some studies suggested that the idiopathic risk factors may include obstetric complications, fetal hypoxia, maternal or paternal age, gestational bleeding, gestational diabetes, prenatal diet, and medication [123,124]. A growing amount of evidence has indicated that mitochondrial dysfunction plays an important role in the development of ASD. ASD is also associated with redox abnormality and oxidative stress [122]. A few studies have suggested that children with autism have limited availability of thiol and reduced glutathione (GSH) storage capacity, resulting in decreased detoxification, increased oxidative stress and DNA damage, and chronic inflammatory responses [125,126,127,128].

Some studies support a significant relationship between ASD and metal exposure [11]. Arora et al. measured the tooth-matrix biomarkers from twin samples, and found that the absorption of Mn and Zn decreased while Pb increased in the ASD patients. In addition, Mn and Pb were also associated with the severity and characteristics of ASD [129]. A systematic review and meta-analysis concluded that early life iAS exposure is positively associated with ASD, and the relationship between lead exposure and autism risk is controversial [130]. Another recent systematic review and meta-analysis indicated that existing evidence supports significant associations between ASD and Al, Cd, and Hg, respectively [131]. Some data demonstrate that the neurotoxic mechanisms of which metals trigger or accelerate the onset of ASD include oxidative stress, endoplasmic reticulum (ER) stress, and destruction of essential metalloproteins, which further lead to or promote neuroinflammation, excitatory toxicity, and apoptosis [11]. In a mouse model, perinatal Pb exposure significantly reduced the activities of SOD, glutathione peroxidase (GPx), and glutathione-disulfide reductase (GSR) in the hypothalamus, corpora quadrigemina, and corpus striatum [132]. A recent cross-sectional study found that zinc levels in hair were inversely associated with the severity of autism symptoms [133]. Fe deficiency was more common in children with ASD compared to the control group [134], and low serum Fe and ferritin levels may be associated with attention deficit hyperactivity disorder [135].

### 2.5. Amyotrophic Lateral Sclerosis (ALS)

ALS is a devastating motor neuron disorder that typically affects men and women between the ages of 50 and 60, and is characterized by progressive muscle weakness, paralysis, and death within a few years of onset [94]. The majority of cases are sporadic, but about 10% are inherited [17]. Disruption of mitochondrial structure, dynamics, bioenergetics, and calcium buffering has been considered to be directly involved in the pathogenesis of ALS [1,17]. Many identified ALS genes play a role in mitochondrial-related functions; for example, superoxide dismutase 1 (SOD1), ALS2, fused in sarcoma/translocated in sarcoma (FUS), VAMP-associated protein type B and C (VAPB), and open reading frame 72 on chromosome 9 (C9orf72). Evidence gathered from patient studies as well as in vitro and in vivo studies strongly reveals that mitochondrial dysfunction is a core event in ALS [1]. Indeed, increased levels of ALS-associated mutant mitochondrial SOD1 may lead to mitochondrial aberrations in ALS [59]. SOD1 mutations are the most common mutation found in ALS, present in about 20% of familial cases and about 2% of overall cases [136]. Mutant SOD1 has been reported to be involved in pathogenesis of ALS through oxidative stress, ER stress, glutamate toxicity, mitochondrial dysfunction, axonal transport disruption, extracellular toxicity, and amyloid aggregation [137]. Under normal circumstances, mitochondria convert 1–3% of oxygen molecules into superoxide radicals, which are later eliminated by SOD1. Thus, in the absence of SOD1, the slowed dismutation process will lead to oxidative stress [138]. Expansion of GGGGCC (G4C2) repeats in the C9orf72 is the most common genetic cause of ALS with frontotemporal dementia (C9-ALS/FTD). Increased ROS and mitochondria hyperpolarization have been reported in the fibroblasts of C9-ALS/FTD patients [139]. A recent genetic study releaved that poly (GR), a dipeptide translated from G4C2 repeat transcript, could be inhibited by yeast mitochondrial escape 1-like ATPase (YME1L) and mitochondria-associated noncanonical Notch signaling [140].

The role of heavy metal exposure (such as Pb, Se, Hg, Cd, and Fe) as a risk factor for ALS has been studied [141]. A recent systematic review and meta-analysis indicated that environmental/occupational Pb exposure was positively proportional to the risk of ALS [142]. In vivo studies have shown that Hg accumulates in the nervous system and damages the axons of motor neurons, consistent with the typical pathological changes of neuron degeneration in ALS [143]. Pb and methyl-mercury (MeHg) can induce ALS-linked TAR DNA-binding protein 43 (TDP-43) accumulation in neurons [144]. Beqollari et al. found that exposure to low doses of MeHg could accelerate the onset of ALS in a SOD1-G93A mouse model probably through glutamate-mediated excitotoxicity [145]. High concentrations of Cd have been detected in blood, cerebrospinal fluid (CSF), and gray and white matter in ALS patients [146,147,148]. Interestingly, a case-report study of ALS showed that Cd disrupted the blood–brain barrier (BBB), decreased SOD1 levels in brain, and enhanced the glutamate excitability in glial cells [138]. In addition, higher Mn contents in the CSF of ALS patients have also been reported, suggesting that the regulation of Mn distribution in human body might play a role in the etiology of ALS [148]. Peters et al. found blood Se and Zn concentrations were negatively correlated with ALS, while blood Cu content was positively correlated with ALS [149]. Se has a protective effect on ALS, which may be related to the protective antioxidant mechanism [150]. Besides, Cu and Zn may play a more direct role in the pathogenesis of ALS, because both are cofactors for cytosolic SOD1. Most polymorphisms lead to misfolding of the SOD1 monomer, reducing its affinity for Zn and exposing the Cu binding site, and this conformational change leads the enzyme to generate rather than detoxify ROS [151,152]. Free Fe level was also higher in the CSF of ALS patients compared to controls [153,154], which may increase iron redox activity and ROS production [155].

## 3. Molecular Mechanisms of Metal-Induced Mitochondrial Dysfunction

### 3.1. Arsenic (As)

As, a widely distributed toxic metalloid, is a risk for about 200 million people in more than 24 countries around the world [156,157]. It can be absorbed through skin, digestive tract, and inhalation. After absorption, As can be distributed to various organs, including kidney, lung, liver, and spleen in the animal and human bodies [158,159]. More seriously, As can enter the central nervous system (CNS) through the BBB and accumulate in different brain regions [160,161,162]. In vivo studies showed that excessive exposure to As induced neuronal apoptosis, which interrupted the neurodevelopment and cognitive functions of rats [163,164,165]. Epidemiological studies in rural-dwelling adults and elders also show that As (3–15 µg/L) levels in water negatively correlated with the scores of cognitive performance and memory, indicating that As is a neurotoxic metalloid [166], which also acts as a risk factor for AD [33,167,168,169]. However, the mechanisms of As-induced neurotoxicity remain unclear.

To date, As-induced neurotoxicity has been related to Aβ overproduction [32,170], inflammatory responses [171,172], thiamine deficiency [173], oxidative stress, disruption of neurotransmitters [163,171], cytoskeletal gene expression, mitochondrial dysfunction, and disruption of acetyl cholinesterase activity [166,167,174]. Among them, mitochondrial dysfunction has been demonstrated to play a key role in As-induced neurotoxicity. Several in vitro studies have shown that As may induce adverse effects on mitochondrial functions. For example, Haga et al. [175] suggested that aggregated mitochondria were found in A172 cells after 50 μM arsenic trioxide (As_2_O_3_) treatment for 8 h. Subsequently, other investigators also suggested that sodium arsenite (NaAsO_2_) or As_2_O_3_ treatment induced mitochondrial dysfunction via increasing intracellular Ca^2+^ levels, mitochondrial membrane potential (MMP), or calpain 1 levels in N_2_A cells [176], SHSY-5Y cells [177], and primary astrocytes [178], as well as rats’ primary neuronal cells [179]. Moreover, in vivo studies have also verified the critical roles of oxidative stress and mitochondrial dysfunctions in As-induced neurotoxicity [180,181].

It is well known that the mitochondrion is the main source of ROS formation, as well as a major target of ROS [182]. Oxidative stress is closely related to mitochondrial dysfunctions induced by As. Yadav et al. [183] showed that the activities of oxidative stress marker enzymes MnSOD and CAT were decreased by As in the mitochondrial fraction of different brain regions (including striatum, hippocampus, and frontal cortex) of rats via increasing ROS, and lipid peroxidation after exposure to NaAsO_2_ for 28 days [181,183]. Similar results were found in sub-chronic As exposure studies done by other investigators which indicated that MnSOD, CAT, Gpx, GR, and GST activity were decreased in the mitochondrial fraction of rat brain [184,185]. Moreover, various studies suggested that As directly impaired the mitochondrial respiratory system via oxidative stress. Dwivedi et al. [180] indicated that As caused oxidative stress which in turn inhibited the activities of complexes I, II, and IV in the mitochondria of rat brain. These results have been corroborated by other labs [181,185]. Furthermore, excessive As exposure disrupted oxidative phosphorylation, and thus interrupted the ATP synthesis and mitochondrial respiration in the mitochondria of the brain [180,186]. Consistent with these results, sub-chronic exposure to low levels of As has been shown to decrease gene expression of the mitochondrial complexes II, IV, and V in mice brains [187,188]. All of the above-mentioned studies suggested that the mechanisms of oxidative stress involved in As-induced mitochondrial dysfunctions play a pivotal role in As-induced neurotoxicity.

In summary, these studies suggest that the mitochondrial dysfunction in the CNS is the most important mechanism of As-induced neurotoxicity. It includes impairments of Ca^2+^ homeostasis [177,189], abnormal mitochondrial dynamics [190,191], and changes in membrane potential and permeability [174,192], which induces neuronal injuries via the mediating mitochondria-dependent pathway.

### 3.2. Aluminum (Al)

Al is a ubiquitously distributed metal on the earth, and it can be easily absorbed via skin contact, inhalation, and ingestion. Al sulfate has been ubiquitously used for water purifying, food processing, and the medicine and pharmaceutical industry, which ensure its presence in human bodies [193]. An increasing number of studies have shown that Al could accumulate in various mammalian organs, including bone, kidney, lung, liver, spleen, and brain [194,195,196]. Growing evidence has also suggested that Al accumulations in various brain regions may cause neurotoxic symptoms and learning impairment [196,197]. Studies in rodents indicated that chronic Al exposure led to Al accumulation in the hippocampus and caused neurobehavioral impairment [198,199,200]. Other studies also reported that Al caused neurofibrillary degeneration [197]. Altmann et al. showed that the impairment in cerebral function may be related to the concentrations of Al in the contaminated water [201]. Additionally, epidemiological studies suggested that Al has been considered as a potential risk factor in the development of neurodegenerative diseases, such as AD [196,202], PD [203,204], and ALS, etc. [205,206,207].

Several studies have proposed that mitochondrial dysfunction may play a critical role in the toxic effects of Al, including neurotoxicity [197,208]. Rao et al. [209] have shown that the ROS formation and mitochondrial respiratory activity, as well as glutathione depletion, were increased in the glial cells after being treated with Al for 24 h. Other groups have also depicted that Al exposure increased ROS formation and impaired the cytochrome c oxidase, which impaired mitochondrial functions in various neuronal cell types, including PC12 [210,211,212], SH-SY5Y neuroblastoma cells [213,214], and rat and cerebellar granule neuronal cells [42,215]. Mitochondrial dysfunction was also observed in in vivo studies [216,217]. Acute exposure to 50 μM Al maltonate via intracisternal injection caused the release of cytochrome c (cyt-c), accompanied by decreased Bcl-2, upregulated Bax, p53, and caspase-3, and DNA fragmentation in the mitochondria of rabbit brain [218]. Subsequently, Kumar et al. also reported that sub-chronic Al exposure for 12 weeks resulted in elevated ROS generation, and decreased ATP synthesis and cytochrome levels in a rat’s brain, which implied disruption of mitochondrial function [219]. In addition, their other study also suggested that Al exposure decreased MnSOD and aconitase activities in different regions of the rat brain [220]. Additionally, transmission electron microscope results showed that Al exposure caused mitochondrial swelling and vacuolization structures, and thus increased the diameter of mitochondria in the hippocampus nerve cells of mice and rats [208,219]. Finally, Al exposure upregulated the autophagy-related proteins LC3-II and Beclin-1, while downregulating p62 expression, suggesting that Al-induced learning and memory impairments may be related to mitophagy [208].

Recently, oxidative stress and mitochondrial disorders have been suggested as major targets for Al-induced neurotoxicity. For example, quercetin has shown protective effects on Al-induced mitochondrial swelling and chromatin condensation in rat hippocampus [221]. Naringin also has protective effects on memory impairment of sub-chronic Al-exposed rats via preventing the activations of mitochondrial oxidative damage in the brain [222]. Subsequently, *Centella asiatica*, which has antioxidant properties, was shown to ameliorate memory impairment and the activation of oxidative stress and decrease mitochondrial enzyme activity in the hippocampus and cerebral cortex induced by Al [223]. In addition, some other natural compounds also have been shown to have neuroprotective effects on Al-induced neurotoxicity, such as crocin, curcumin, and polyphenols [197,224,225]. These studies indicate that inhibition of oxidative stress and mitochondrial dysfunction may be a therapeutic strategy to prevent the neuronal injuries induced by Al.

### 3.3. Copper (Cu)

Cu is an essential trace metal for human health. Cu takes part in many cellular enzymatic activities, including energy production, redox balance, and neurotransmitter biosynthesis [226]. An adequate amount of copper is critical for the maintenance of redox balance in the mitochondria [227]. The mitochondria are both a regulatory hub for Cu homeostasis and a target of Cu toxicity [228]. For example, Cu is required for metallation of the catalytic core of cytochrome c oxidase, a mitochondrial metalloenzyme in the respiratory complex chain [229]. However, overload of mitochondrial Cu is detrimental to the function of respiratory complexes, leading to elevation of ROS and mitochondria dysfunction. Wilson’s disease is a genetic disorder caused by excessive mitochondrial copper in the liver [227].

Brain mitochondria are particularly sensitive to the detrimental effects of Cu [230]. Compared to the mitochondria in the liver, kidney, and heart, brain mitochondria are susceptible to elevated levels of Cu, which attacks free thiols in large molecules that are indispensable for maintaining neuronal cell function [230]. The membrane potential, efficiency in ATP production, and structural integrity of brain mitochondria were prone to damage caused by excessive Cu [230]. Chronic Cu exposure led to spatial memory impairment that was associated with mitochondrial damage in the hippocampus [231]. Specifically, beta-amyloid-induced memory deficit in rats is exacerbated by Cu exposure. Meanwhile, analysis of isolated mitochondria from rat hippocampus following Cu exposure demonstrated a significant decline in mitochondria health, including increased lipid peroxidation and glutathione oxidation [231]. Mishandling of Cu in the mitochondria has been linked to age-related neurodegenerative disorders [232,233,234]. In a mice model of AD, a proteomics study showed that low levels of Cu exposure (0.13 ppm, 2 months) induced deficits in mitochondrial dynamics, leading to increased H_2_O_2_ production and reduced cytochrome oxidase activity [232]. Common biochemical characteristics of PD include accumulation of iron and diminished Cu content in degenerated brain regions. The disruption of Cu metabolism was believed to be involved in the pathological process in loss of catecholamine neurons [233]. Additionally, in a 6-hydroxydopamine (6-OHDA)-induced-PD model, Cu exposure increased oxidation of 6-OHDA, resulting in an increase in the rate of p-quinone formation and H_2_O_2_ accumulation. In the same model, the 6-OHDA-induced lipid peroxidation and protein oxidation were potentiated by Cu exposure [234].

Mitochondrial dysfunction following chronic Cu exposure involves oxidative stress, collapse in mitochondrial membrane potential, depletion of GSH, comprised function of respiratory complexes, reduction in APT production, and structural damage to the mitochondria [230,231]. Experimental evidence showed that free protein thiols in the mitochondria are potential toxic targets of Cu [230]. GSH supplementation attenuated Cu-induced lipid peroxidation but failed to protect oxidized thiols [234]. In addition, the induction of the mitochondrial permeability transition (MPT) was associated with Cu-induced astrocytic injury [235]. Furthermore, mitochondrial health in the hippocampus is a potential in vivo target of Cu. A recent study showed that mitochondrial biogenesis and respiratory function were impaired in the hippocampus of mice chronically exposed to CuCl_2_ [232].

### 3.4. Cadmium (Cd)

Cd is a heavy metal that has no nutritional roles for humans. Cd-induced cellular damage is largely mediated by disruption of mitochondrial activity [236]. Elevation of ROS in the mitochondria and induction of mitochondria-derived apoptosis signaling are involved in Cd-induced neurotoxicity [237,238]. Mitochondrial protection afforded by antioxidants can attenuate Cd-induced neuronal damage [239].

An elevation in protein and lipid peroxidation, decrease in antioxidant capacity, and structural damage to the mitochondria were shown in the brains of rats chronically exposed to Cd [240]. The structural stability of mitochondria-associated ER membranes (MAMs) is critical for the proper function of the mitochondria. Recent studies show that MAMs are not only the physical bridge to facilitate communication between the ER and mitochondria, but they are also indispensable for cellular homeostasis processes such as autophagy, lipid metabolism, and Ca^2+^ transport [241]. Cd exposure induced increased production of ROS in the mitochondria, leading to impairment of MAMs [242]. The shapes of mitochondria are subjected to transformations in response to cellular stress, which is driven by two closely related processes: mitochondrial fusion and fission. Mitochondrial fusion and fission are required for proper intracellular distribution and quality control of the organelle [243]. Mitofusin 2 (Mfn2) is a mitochondrial outer membrane-localized GTPase that is essential for mitochondrial fusion. Cd-induced neuronal necroptosis was associated with ROS-induced S-glutathionylation of Mfn2 [242]. Increased ROS levels are detrimental to the activity of key enzymes involved in lipid metabolism. Cd exposure altered the lipid profile in a rat brain, resulting in an increased level of cholesterol (CHL) in the mitochondria [244]. Furthermore, Cd exposure promotes lipid peroxidation (LPO), which is mediated by the increased level of oxygen free radicals [245]. The mitochondria are both a storage site for cellular calcium ions and regulators for calcium ion homeostasis. Cd can competitively bind receptors and ion channels that regulate calcium ion influx, modulating calcium-dependent cellular activity [246]. The Ca^2+^/calmodulin-dependent protein kinase II (CaMK-II) regulates cytoskeletal dynamics and apoptotic cell death. Recent advances show that CaMK-II mediates the effects of Cd exposure on actin depolymerization microtubules and cadherin junctions, which are the underlying mechanisms of Cd-induced cytoskeletal disruption and alterations in cellular morphology [246]. Nutritional trace metals, such as Zn and Se, can mitigate Cd-induced mitochondrial toxicity. For example, in a cellular toxicity model of PC12 cells, Cd exposure led to depletion of cellular GSH and oxidative damage to the mitochondria, which can be attenuated by Zn supplementation [247]. Additionally, Se supplementation suppressed Cd-induced oxidative stress and the mitochondrial apoptosis pathway [237].

### 3.5. Mercury (Hg)

Mercury is a naturally occurring element that is found in various inorganic and organic forms [248,249]. Both organic and inorganic mercury are neurotoxic. Methylmercury (MeHg) is of special concern as it is an ubiquitous environmental contaminant and its consumption in fish can lead to a devastating neurological disorder, referred to as Minamata disease [250]. Numerous studies have shown that mercury causes brain mitochondrial dysfunction, playing a key role in Hg-induced brain damage and neurological disorders.

As early as 1974, Chang and Hartmann found that mercury was present both in neurons and in glia after MeHg or mercuric bichloride (HgCl_2_) administrated to rats orally or subcutaneously [251]. Notably, mitochondria accumulate mercury, mostly because of their abundance of thiol (–SH) groups. Although mercury initiates multiple additive or synergistic disruptive effects, a key mechanism of disruption of mitochondrial function is associated with the production of ROS. HgCl_2_ and/or MeHg exposure enhance ROS formation in the CNS, evidenced by both in vivo [252] and in vitro models, including primary rat cortical neuron [253], rat cortical astrocyte [254,255], cerebellar granule neurons and astrocytes [256], and microglia [257], as well as in mixed primary neuron–astrocyte culture [258]. ROS overgeneration leads to consequent oxidative stress [259] and mitochondria-mediated apoptosis. For example, MeHg exposure results in cytochrome c release, caspase-3 and caspase-9 activation, and apoptosis-induced factors (AIF) increase in primary rat cortical neuron [253]. Mitochondria-mediated apoptosis in brain cells is secondary to alteration of mitochondrial membrane potential (MMP) and transition of mitochondrial permeability [260], which have been observed in neuron/astrocyte mixed-culture [258] and astrocyte mono-culture [261,262] after mercury exposure. In addition, the mitochondrial dysfunction evoked by mercury was correlated with damage in mitochondrial bioenergetics. Mercury has been found to act as an inhibitor of the enzymatic activities of mitochondrial respiratory complexes, impairing ATP synthesis in rat hippocampal mitochondria [263]. MeHg exposure reduced GSH levels in astrocytes, increasing the vulnerability to oxidative stress [264]. Apart from a series of biochemical impairments in mitochondria induced by mercury exposure, pathological changes in mitochondrial morphology have also been demonstrated. Li et al. [265] found that a low dose of mercury, lead, and cadmium caused dose-dependent mitochondrial depletion, as well as ridge and matrix dissolution in the hippocampal neurons of rats. Additionally, an in vivo study observed that MeHg induced mitochondrial swelling in the hippocampus of MeHg-exposed F1 generation rats, and enlarged and fused mitochondria in mice cerebral cortex [263].

Dreiem and Seegal [266] found that antioxidant Trolox significantly reduced MeHg-induced ROS, while failing to restore mitochondrial function in rat striatal synaptosomes. The authors revealed that MeHg increased mitochondrial calcium levels, which are fundamental to mitochondrial function. If mitochondria take up too much Ca^2+^, it delays the rise in cytoplasmic Ca^2+^ [267] and the opening of the MPT pore, which may promote the release of cytochrome c and other pro-apoptotic factors, culminating in apoptosis [268]. The modulatory effect of cellular calcium homeostasis by MeHg in mouse spinal motor neurons was also found [269]. In addition, proteomic analysis revealed that many mitochondrial proteins were deregulated by mercury exposure in primary mouse cerebellar granule neuron and astrocytes [256,270], as well as in rat hippocampus [271], thus impairing mitochondrial function associated with cellular metabolism and energy production.

### 3.6. Lead (Pb)

Pb is an environmentally abundant metal pollutant with human exposure mainly through air inhalation and food and water intake. Pb is a strong toxicant for the developmental CNS [272,273]. Pb intoxication in children, even at low doses, is found to impair learning and memory and affect cognitive functions and intellectual development [274,275]. The brain is the primary target of Pb toxicity. Mitochondria play a key role in Pb-induced impairment of nervous system function.

An in vivo study found that the activity or levels of several mitochondrial enzymes were inhibited by Pb exposure. For example, lead acetate (PbAc) exposure in drinking water decreased aldehyde dehydrogenase (ALDH2) expression in brain nucleus accumbens [276], and PbAc exposure from postnatal day 1 (PND1) through PND21 in drinking water of the mother significantly decreased offspring activity of mitochondrial monoamine oxidase (MAO) in all brain regions, including cerebral cortex, hippocampus, and cerebellum, in a dose- and age-dependent manner [277], attributed to the high affinity of Pb for the -SH groups in enzymes, consequently damaging mitochondrial activity and function. In addition, pre- and neonatal exposure to a low dose of Pb (Pb concentration in whole blood < 10 μg/dL) induced synaptic ultrastructural abnormalities in mitochondria including elongated, swollen, and shrunken changes in mitochondria [278], indicating the mitochondrial morphological disruption induced by Pb. Mitochondria-mediated apoptosis has also been shown in Pb-induced neuronal death. PbAc intoxication caused cognitive dysfunction and anxiety-like behavior, along with altered Bax/Bcl-1 expression and increased cytochrome c release from mitochondria in rat brain [279]. In addition, (CH_3_COO)_2_Pb exposure induced apoptosis via the mitochondrial pathway in embryonic neurocytes isolated from chicken [280]. Similarly, the combined treatment (As+Cd+Pb) in individual lethal concentration (LC)-5 induced a toxic effect on C6-glioma cells derived from rat glioma, via mitochondria-mediated apoptosis, including caspase-9 activation and Bax/Bcl-2 changes [281]. Notably, Zhu et al. found that MPT pore opening plays an important role in Pb-induced neurotoxicity. In SH-SY5Y cells, PbAc exposure significantly impaired mitochondrial function, evidenced by ATP decrease, MMP collapse, ROS production, mitochondrial apoptosis, and morphology changes (swelling and rupture). PbAc treatment significantly increased the protein level of Cyp D, a component of MPT, and induced MPT pore opening in both PC12 and SH-SY5Y cells. Inhibitor of Cyp D significantly reversed mitochondrial damages and cell death induced by Pb [282].

### 3.7. Zinc (Zn)

Zinc is an essential trace element that is required for the function of numerous enzymes and DNA-binding transcription factors. Excess zinc influx has been manifested to play a role in neuronal damage and death associated with traumatic brain injury, stroke, seizures, and neurodegenerative diseases [283,284]. Mitochondria have been identified as targets of the neurotoxic effects of zinc by reducing ATP production and increasing ROS.

Zinc exposure reduced the cellular nicotinamideademine dinucleotide (NAD+) in cultured mouse cortical neurons, followed with a progressive loss of ATP levels and subsequent cell death [285,286,287], indicating the potential inhibition of mitochondrial respiration enzyme. Indeed, several mitochondrial enzymes, including α-ketoglutarate dehydrogenase, NAD+-dependent isocitrate dehydrogenase, succinate dehydrogenase, and cytochrome c oxidase, have been demonstrated to be inhibited by zinc exposure in liver mitochondria [288,289]. Notably, by using bovine heart mitochondria, complex III, specifically the bc 1 complex, was identified as the site of Zn^2+^ binding and inhibition [290,291]. ROS generation has been found to be critical in zinc-induced neurotoxicity, demonstrated in diverse brain cell models [292,293]. As mitochondria are an important source of cellular ROS production, the influx of Zn^2+^ through Ca^2+^-permeable AMPA/kainate channels also triggers rapid mitochondrial depolarization, leading to prolonged production of mitochondrial superoxide in cortical neurons [294].

In addition, several other mechanisms have been involved in the zinc-induced mitochondrial dysfunction. For example, extracellular zinc application stimulates the Ras/MEK/ERK pathway, which leads to zinc-induced mitochondrial dysfunction and consequent cell death in rat neurons [295]. An immediate early transcription factor, egr-1, was found to act downstream of ERK 1/2 to induce neuronal death after zinc exposure [296]. Furthermore, elevated intra-neuronal zinc impairs mitochondrial trafficking without altering morphology, which was restored by PI3k inhibitors, suggesting the role of PI3k activation in zinc-inhibited mitochondrial movement in neurons [295]. Apart from the adverse effects on neurons and glia, zinc overload also critically induced ROS formation in mitochondria and degradation of mitochondrial network in cerebral microvessels, which were mediated through Drp-1-dependent mitochondrial fission pathway, thus contributing to increased permeability of the BBB after cerebral ischemia.

Not only zinc overload, but also zinc defficiency, may impair neurological functions [297] and cause neuronal apoptosis via an intrinsic (mitochondrial) pathway in human neuroblastoma IMR-32 cells and primary rat cortical neurons [298]. Researchers have identified that the transposition of phosphorylated p53 into the mitochondria mediated zinc deficiency-induced mitochondrial alterations and apoptosis in neuronal precursor cell (NT-2 cell line) [299].

### 3.8. Iron (Fe)

Iron is a crucial trace metal for life and is the most abundant transition metal in the brain. It acts as a catalytic center for multiple enzymes and supports many elementary biological processes, including DNA synthesis and repair, oxygen transport, mitochondrial respiration, and neurotransmitter metabolism. Oxidative stress, iron deposition, and mitochondrial dysfunction have been considered as hallmarks of many neurodegenerative diseases, including PD, HD, and AD [300,301], and a positive feedback loop among these three factors seems to exist in neurological disorders.

Upregulation of cellular redox-active iron is directly related to increased ROS and with changes in intracellular reduction potential [302,303]. In the presence of H_2_O_2_, which is mainly produced by mitochondrial ETC, Fe^2+^ generates hydroxyl radicals (OH) via the Fenton reaction. The hydroxyl radical is considered to be one of the most reactive substances in biological systems because its reaction rate is limited only by its diffusion. This free radical can attack proteins, DNA, and lipid membranes, thus disrupting mitochondrial function and cellular integrity, and eventually leading to oxidative stress and cell apoptosis [304]. Iron overload promotes the production of mitochondrial ROS in SH-SY5Y cells, in an AMP-activated protein kinase (AMPK)-dependent manner [305], and caused ATP production defects, mitochondrial complex I inhibition, and mitochondrial apoptosis in primary cortical neurons [306]. In addition, mitochondria-targeted iron chelators showed protective effects against mitochondrial oxidative damage and neuronal death, both in rotenone-treated SH-SY5Y cells and the dopamine neurons from MPTP-intoxicated mice, which indirectly suggested that iron accumulation in mitochondria induced mitochondrial oxidative damages in neurons and consequent cell death [307]. Moreover, iron overload may induce Drp-1-dependent mitochondrial fragmentation by upregulating intracellular calcium. Lee et al. [308] found that in ferric ammonium citrate (FAC)-stimulated HT-22 hippocampal neuron cells, mitochondria were fragmentated by dephosphorylation of Drp1 (Ser637) and apoptotic neuronal death was increased. Notably, FAC-induced iron overload leads to intracellular calcium elevation and further activation of calcineurin, while inhibition of Ca^2+^ signals related to calcineurin prevents iron overload-induced mitochondrial fragmentation and neuronal cell death. Redox-sensitive ryanodine receptor (RyR)-mediated Ca^2+^ release also was shown to underlie the iron-induced mitochondrial fission in primary hippocampal neurons [309].

Recently, a new iron-dependent programmed cell death, namely ferroptosis, has been found to be a main driver of many neurodegenerative diseases. It is characterized by the accumulation of lipid peroxidation products and lethal ROS derived from iron metabolism and can be pharmacologically inhibited by iron chelators. Although the detailed mechanism by which iron overload promotes ferroptosis has yet to determined, it is reasonable to hypothesize that iron overload may drive the generation of hydroxyl radicals, which further react with liposomes to produce lipid peroxidation products and cause mitochondrial dysfunction, and eventually ferroptosis [310,311,312]. Although mitochondria have been shown to be vital regulators of iron homeostasis and ferroptosis in neurodegenerative diseases [313], more direct evidence targeting iron overload, mitochondrial dysfunction, and ferroptosis is still required. The mitochondria are also the site for the synthesis of iron–sulfur cluster biogenesis (ISCs) and heme prosthetic groups. There is evidence that mitochondrial ISC assembly defects may cause iron overload and consequent negative effects on cellular or mitochondrial function [314,315].

Therefore, iron accumulation induced by direct excessive iron exposure or secondary to iron overload has been demonstrated to play an important role in neurological diseases, via impairing mitochondrial function and inducing oxidative stress. Targeting chelatable iron and the consequent ROS, especially in mitochondria, appear as possible therapeutic options for age-related neurodegenerative conditions [316].

### 3.9. Manganese (Mn)

Mn is the 12th most abundant mineral element in the earth crust, and is both nutritionally essential and toxic in excess. Mn is an essential metal for normal growth, development, and cellular homeostasis, as well as a cofactor for multiple enzymes; for example, Mn-superoxide dismutase (Mn-SOD), pyruvate carboxylase, arginase, and glutamine synthase (GS). Manganese preferentially accumulates in tissues rich in mitochondria [317,318], and it is taken up by brain mitochondria via mitochondria Ca^2+^ uniporter [319].

Mn is known to induce mitochondrial dysfunction in the nervous system [320], including the inhibition of the enzymes of the tricarboxylic acid (TCA) cycle in human neuroblastoma (SK-N-SH) and astrocytoma (U87) cells [321] and a reduction in the activities of ETC in rat primary striatal neurons [322] and in PC12 cells [323], ultimately resulting in ATP depletion [324,325,326] and mitochondria-mediated apoptosis [327,328,329]. Notably, these mitochondrial impairments have been found to be rescued by some antioxidants [324,325,330], indicating that oxidative stress is primarily involved in the mechanism of Mn-induced mitochondrial dysfunction.

Another cause of mitochondria-mediated apoptosis induced by Mn exposure is the induction of the MPT [331]. This process causes unrestricted proton movement across the inner mitochondrial membrane, resulting in mitochondrial swelling, mitochondrial membrane potential destruction, further production of ROS, and cellular apoptosis [324,332].

## 4. Conclusions

Long-term exposure to various metals, both essential and non-essential, has become increasingly common among the public as accelerated human activities release excess metals into the environment. Studies over the past several decades have greatly improved understanding of the neurodegenerative diseases associated with metals exposure and refined the molecular mechanisms of metal-induced nerve cell damage. Excessive exposure to both essential and non-essential metals may cause neurotoxicity, but deficiency in some essential metals, such as Zn and Fe, can also aggravate neurodegenerative diseases. As reviewed herein, metals may play a role in some neurodegenerative diseases, including AD, PD, HD, autism, and ALS, all of which rely on similar intracellular mechanisms, including metal dyshomeostasis, mitochondrial dysfunction, oxidative stress, and protein aggregation. Of all organelles, mitochondria produce the most intracellular ROS [102]. Excessive production of ROS and/or inhibition of the antioxidant system in mitochondria may cause oxidative stress, damage the mitochondrial structure, and induce apoptosis, which plays an important role in neurodegeneration. Upstream mechanisms of ROS generation are nonetheless not well characterized, and additional studies are required. For example, Nrf2 is known to be activated by methylmercury, copper, and other metals, but other Nrf2-independent means for ROS mitigation have also been described.

To date, most studies have focused on the neurotoxic mechanisms of single metals; however, in real life, the exposure environment of human metal exposure is complex, and metals may interact with each other and influence their homeostasis. It is therefore imperative to further explore the effects of metal mixtures in the etiology of neurological disorders.

## Data Availability

Not applicable.
